# Who is the Usual Suspect? Evidence of a Selection Bias Toward Faces That Make Direct Eye Contact in a Lineup Task

**DOI:** 10.1177/2041669517690411

**Published:** 2017-02-01

**Authors:** Jessica Taubert, Celine van Golde, Frans A. J. Verstraten

**Affiliations:** The School of Psychology, The University of Sydney, Sydney, Australia; The National Institute of Mental Health (NIMH), MD, USA; The School of Psychology, The University of Sydney, Sydney, Australia; The School of Psychology, The University of Sydney, Sydney, Australia; Helmholtz Institute, Utrecht University, Utrecht, The Netherlands

**Keywords:** misidentification, face perception, legal procedures, eye gaze direction, eyewitness identification, applied visual science

## Abstract

The speed and ease with which we recognize the faces of our friends and family members belies the difficulty we have recognizing less familiar individuals. Nonetheless, overconfidence in our ability to recognize faces has carried over into various aspects of our legal system; for instance, eyewitness identification serves a critical role in criminal proceedings. For this reason, understanding the perceptual and psychological processes that underlie false identification is of the utmost importance. Gaze direction is a salient social signal and direct eye contact, in particular, is thought to capture attention. Here, we tested the hypothesis that differences in gaze direction may influence difficult decisions in a lineup context. In a series of experiments, we show that when a group of faces differed in their gaze direction, the faces that were making eye contact with the participants were more likely to be misidentified. Interestingly, this bias disappeared when the faces are presented with their eyes closed. These findings open a critical conversation between social neuroscience and forensic psychology, and imply that direct eye contact may (wrongly) increase the perceived familiarity of a face.

## Introduction

Face perception is the most reliable means of accessing a person’s identity without the use of automated technology, such as iris or fingerprint scans. Yet, there are large individual differences in our ability to recognize faces (Bowles et al., 2009; Russell, Duchaine, & Nakayama, 2009) and particular difficulties are associated unfamiliar faces (Hancock, Bruce, & Burton, 2000). For instance, when Bruce et al. (1999) asked participants to select a target face from an array of 10, in a classic lineup paradigm, they were only able to perform accurately in 70% of trials, despite optimal viewing conditions. Moreover, in that particular study, the photographs and video frames used were taken on the same day, enabling the use of identity cues from external features, such as hairstyle (Bruce et al., 1999). More recently, Megreya, Sanford, and Burton (2013) reported that, when photographs were taken months apart, recognition accuracy suffered even more.

However, despite the overwhelming evidence indicating how difficult unfamiliar face matching is (Bruce et al., 1999; Hancock et al., 2000; Megreya & Burton, 2006; Megreya et al., 2013), person-to-ID matching is commonly used to protect citizens against identity theft and to investigate crime. Kemp, Towell, and Pike (1997) investigated fraud detection among a group of experienced supermarket cashiers tasked with matching a customer’s face with a picture printed on a credit card. The authors reported that in instances where the identities did not match but the photograph resembled the card bearer, cashiers only correctly rejected the identities in 36% of trials, leading to an overall error rate of over 60%. Even in easier trials, where the card bearer did not resemble the photograph, the error rate remained over 30%. This error rate occurred despite the fact that the cashiers were aware of the study’s objectives and were presumably motivated to perform well (Kemp et al., 1997). Turning to forensic settings such as border control, successfully matching a real person to a photograph is often a matter of national security. However, White, Kemp, Jenkins, Matheson, and Burton (2014) have showed that even trained passport control officers are not better at matching identities than university students. In summary, evidence suggests that, even when the stakes are high and people acquire a tremendous amount of practice, unfamiliar face recognition is a painfully difficult task.

### Present Study

We are interested in uncovering the signals people might be relying on to select a face in a lineup, other than *actual* recognition. Eye gaze direction is thought to be a major sociocommunicative signal among social primates (Langton, Watt, & Bruce, 2000; Leonard, Blumenthal, Gothard, & Hoffman, 2012; Senju & Johnson, 2009). Seeing another person looking directly at you is likely to indicate that their attention is directed at you, while averted gaze implies that the other person's attention is directed toward something else (Kuhn & Benson, 2007; Kuhn & Kingstone, 2009; Ricciardelli, Bricolo, Aglioti, & Chelazzi, 2002). Allocating visual attention to direct eye gaze seems to be the basis for social interaction (Baron-Cohen, 1995; Tomasello & Carpenter, 2007; Tomasello, Carpenter, Call, Behne, & Moll, 2005), and a failure to do so is a symptom of severe social disorders such as Autism (Emery, 2000; Klin, Lin, Gorrindo, Ramsay, & Jones, 2009; Tanaka & Sung, 2016). In regard to person recognition, the direction of a target’s eyes has been shown to enhance or influence memory for faces in old or new decision-making tasks in both adults and children (Adams, Pauker, & Weisbuch, 2010; Farroni, Massaccesi, Menon, & Johnson, 2007; Hood, Macrae, Cole-Davies, & Dias, 2003; Smith, Hood, & Hector, 2006; Vuilleumier, George, Lister, Armony, & Driver, 2005). Interestingly, from a neuroscience perspective, changes in eye gaze and direct eye contact are particularly provocative—modulating activity in visual system (Madipakkam, Rothkirch, Guggenmos, Heinz, & Sterzer, 2015; Mosher, Zimmerman, & Gothard, 2014; Pelphrey, Morris, Michelich, Allison, & McCarthy, 2005; Pelphrey, Viola, & McCarthy, 2004; Perrett et al., 1985; Wicker, Michel, Henaff, & Decety, 1998). Collectively, these studies indicate that we are sensitive to gaze direction, and particularly to direct eye contact, but what information does direct eye gaze convey?

The idea that direct eye contact might signal more to a receiver than the direction of that person’s attention has, so far, only been explored in studies that have examined lie detection, which have yielded inconsistent results (Vrij, Granhag, & Porter, 2010; Vrij & Semin, 1996). In this article, however, we have married this idea with the emerging evidence that unfamiliar face recognition in applied settings is an alarmingly difficult task. Our hypothesis that gaze direction might interfere with face recognition in a lineup task was based on three findings:

1. people find it difficult to recognize faces in a lineup (Bruce et al., 1999; Hancock et al., 2000; Pozzulo & Balfour, 2006; Steblay, Dysart, Fulero, & Lindsay, 2003; Wells, 1993) and, thus, might rely on superfluous information to make their judgment such as gaze direction.
2. People automatically process gaze direction without awareness and irrespective of the task at hand (Kuhn & Benson, 2007; Kuhn & Kingstone, 2009; Ricciardelli et al., 2002) and
3. direct eye gaze has been shown to enhance recognition memory for faces in old or new decision-making tasks (Adams et al., 2010; Farroni et al., 2007; Hood et al., 2003; Smith et al., 2006; Vuilleumier et al., 2005); however, it is unknown how this will affect recognition within a lineup setting.

## Experiment 1

In Experiment 1, participants were required to watch a brief movie of a person talking without the benefit of sound and then determine whether that same person was present in a lineup of four people. For half the trials, the target was present and we manipulated the gaze of these targets in four unique conditions (direct gaze, left gaze, right gaze, and upward gaze) with the expectation that performance in target present trials would be better in the direct gaze condition. Participants could also make two different kinds of errors, they could falsely reject a target present trial or they could misidentify a distractor. The number of misidentification errors that occurred for each distractor type (direct gaze; left gaze; right gaze or upward gaze) was also analyzed to determine whether there was a systematic bias toward faces making direct eye contact.

### Method

#### Participants

All participants were psychology undergraduate students from The University of Sydney. In Experiment 1, we recruited 27 participants in total (12 male; aged between 22 and 34 years of age). The first three participants tested (all female) were given a different set of instructions from the remaining 24 participants, as part of an initial pilot. Instead of testing their recognition memory, their responses were used to confirm that the direct gaze stimuli were the faces perceived as looking directly at them. A power analysis based on a pilot study suggested 16 participants would be sufficient to find evidence of an effect among four target types. We collected eight additional participants because the power analysis was based on accuracy in target present trials and one of our hypotheses referred specifically to an increase in the misidentification error rate (i.e., we wanted to measure variance in the false positive rate across four distractor types). Participants gave their written consent prior to completing the experiment. All participants reported normal or corrected-to-normal vision and were naïve as to the purpose of the study. The research protocol was approved by the Human Research Ethics Committee of the University of Sydney (Project No. 2015/336).

#### Stimuli and procedure

**Figure 1.**
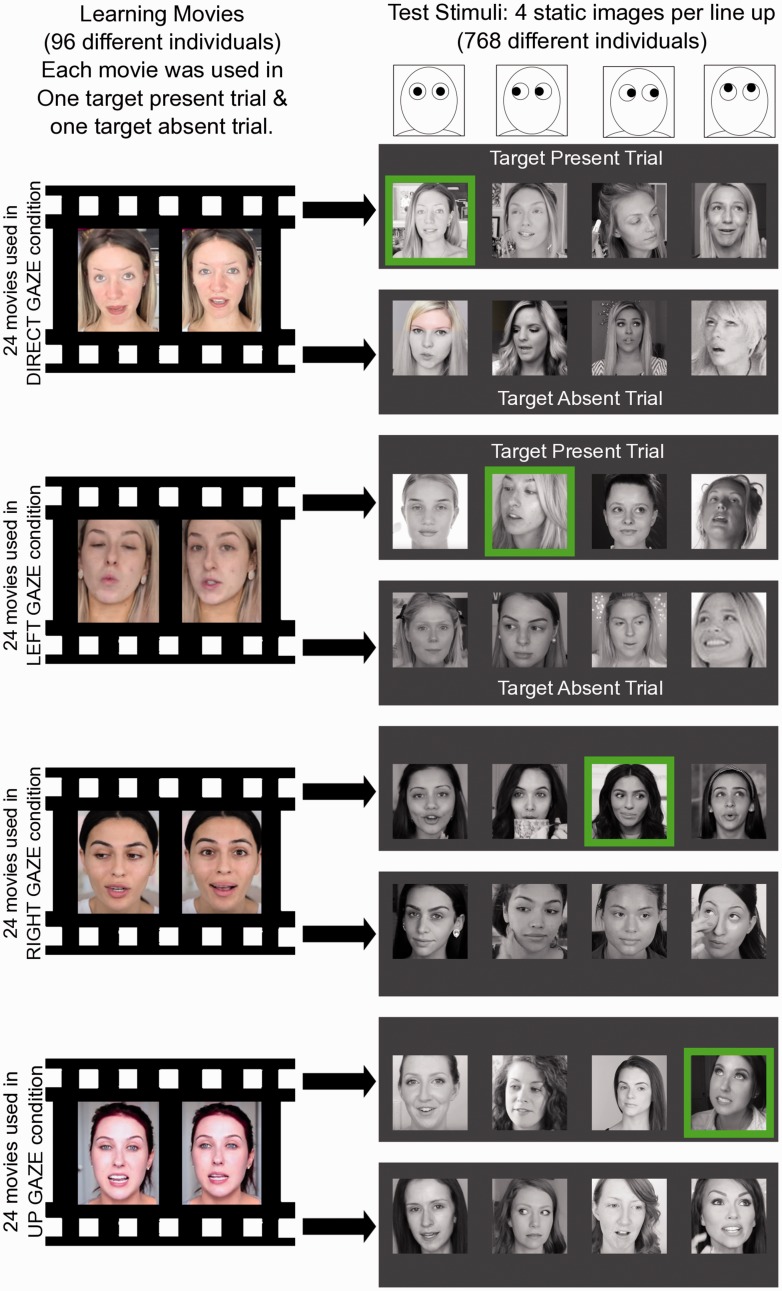
The design for Experiment 1. The green squares indicate the target face in *target present* examples. Otherwise, in *target absent* examples, the correct answer was “not present.”

The stimuli for all three experiments reported here were makeup tutorials originally uploaded on to www.youtube.com. We sourced 96 movies depicting 96 different female tutors, capturing a 5-second period of their monologue at the beginning of their tutorial (without sound). No further editing was performed. These movie files were presented at the beginning of every trial, during the “learning phase,” in the center of the screen on gray background (see Figure 1).

For each of the 96 movies, we sourced a second makeup tutorial (www.youtube.com) that was delivered by the same tutor. This second tutorial varied as much as possible from the first in terms of background, lighting, and clothing. From this second movie file, we took a single frame to serve as the target stimulus. These static images of the actors either depicted them looking directly at the camera (24 faces), looking to the left (their right; 24 faces), looking to the right (their left; 24 faces), or looking up above the camera (see Figure 1). Corresponding distractors (and the stimuli used in the target absent trials) were single images captures from the makeup tutorials, and similar interviews, of other female tutors (672 static images in total; see Figure 1). Although some effort was made to capture different gaze directions without head turns, inevitably these stimuli often also differed in viewpoint. However, we tried to minimize these differences by ensuring that two eyes were always visible (the majority of the differences were ± 30° from the front most viewpoint). These static images were cropped, gray scaled, and placed on a square canvas 300 × 300 pixels in size. The luminance and root-mean-square contrast of each stimulus was adjusted to match the mean luminance and contrast values of the entire image set (768 stimuli in total).

All participants were initially asked if they used the website www.youtube.com to watch movies and whether they had ever watched makeup tutorials on www.youtube.com. Although all of the participants were www.youtube.com users, only 16 admitted to watching makeup tutorials. Importantly, none of the participants reported seeing a familiar face during the experiment.

**Figure 2.**
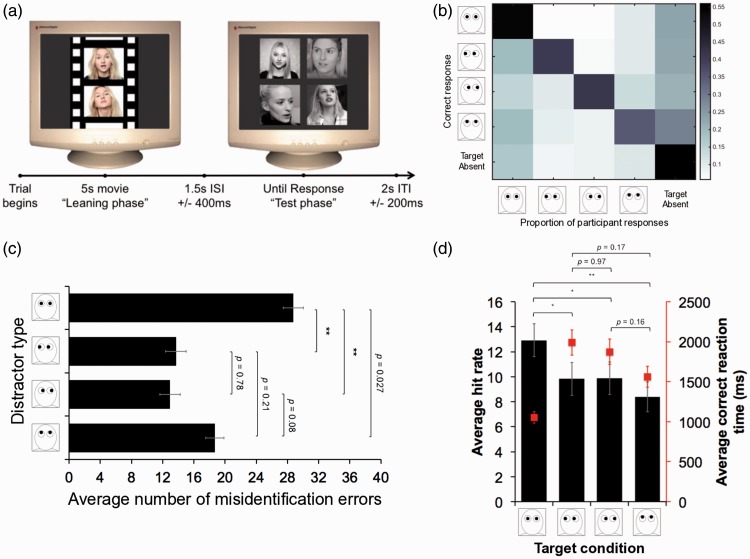
Procedure and results for Experiment 1. (a) Experimental procedure and illustrative stimuli. The example is taken from the direct gaze condition (target is present in top left corner of lineup array). Each array presented participants with four faces (direct gaze; left gaze; right gaze; upward gaze). The location within was randomized. (b) Response profile across all trials. The proportion of participant responses (direct gaze face; left gaze face; right gaze face; up gaze face; target not present) in each of the five target conditions (target present-direct gaze face; target present-left gaze face; target present-right gaze face; target present-upward gaze face; target absent). The dark diagonal reflects overall accuracy and dark columns reflect a bias toward a particular face type that transcends target condition. (c) Misidentification errors. Average number of times each participant incorrectly selected each distractor type (direct gaze face; left gaze face; right gaze face; up gaze face) as the target (error bars = ± *SEM*). ** indicates a *p* value that remained less than .01 after correction. Cohen’s *d* values for repeated factors were calculated for the pairwise contrasts: direct gaze versus left gaze face, *d* = 1.09; direct gaze versus right gaze, *d* = 1.18; direct gaze versus up gaze, *d* = 0.48. (d) Average overall accuracy. Average hit rate in target present trials across participants (error bar = ± *SEM*). Superimposed is the average correct reaction time (secondary y-axis) to verify whether participants were trading speed for accuracy. * indicates a *p* value that was less than .05 after correction and ** indicates a *p* value that was less than .01 after correction. Cohen’s *d* values for repeated factors were calculated for the pairwise contrasts: direct gaze versus left gaze face, *d* = 0.68; direct gaze versus right gaze, *d* = 0.66; direct gaze versus up gaze, *d* = 0.75.

The experimental procedure in Experiment 1 is illustrated in Figure 2(a). Each trial began with a 5-second movie, participants were made aware that they needed to pay attention to the identity of the person in the movie because they would be asked to recognize them in the subsequent test phase. After a interstimulus interval of 1.5 seconds (on average), participants were presented with four faces and asked to decide whether the person from the preceding movie was present or not. The four faces were presented in four positions on the screen (top left; top right; bottom left; bottom right), equally distant from the center (see Figure 2(a)). All four faces were presented at the same size (subtending 5° of visual angle in height) on uniform gray background. The lineups were constructed so that the targets occurred in each of the four positions an equal number of times. If they were present, the participant was required to hit the corresponding key on the keyboard (“7,” “9,” “1,” and “3” representing the four screen positions) or, alternatively, they could press “5” to indicate the person was not present. The lineup was present until a response was recorded, then there was a 2,000 (± 200) ms intertrial interval before the next trial began. We recorded their accuracy and their response speed. Each of the 96 movies were seen twice (serving only once as part of a target present trial) and these 192 trials were presented in a random order.

Participants completed Experiment 1 in a dimly lit curtained booth. The experiment was programmed in MATLAB version R2010a using the Psychophysics Toolbox 3 (Brainard, 1997; Pelli, 1997). The program ran on an Apple Mac Pro (Lion 10.7.5). Participants were seated approximately 57 cm in front of a CRT monitor (18-inch viewable screen size) set at a screen resolution of 1024 × 768 pixels with a refresh rate of 100 Hz. Participant input was recorded using an Apple wired USB keyboard.

### Results

Initially, we ran three participants who completed the same experiment but were instructed to respond differently from the 24 in the main sample. For the first three participants, this was not a test of recognition memory, rather they had to simply indicate, under the same experimental conditions, which of the four faces was looking directly at them (including both targets and distractors; a four alternative force-choice task with no option to indicate none). All three were able to choose the faces in the direct gaze condition without fail.

For the remaining 24 participants, the instructions were to study the face in the learning phase and report whether that person was present in the subsequent lineup. The response profile of participants (Figure 2(b)) indicated a tendency to incorrectly reject in target present trials, but also a bias toward direct gaze faces (and to some extent upward gaze faces). After counting the number of misidentification errors participants made (see Table 1), we assessed which type of distractor was driving the misidentification error rate (Figure 2(c)). A significant one-way repeated measures analysis of variance (ANOVA) comparing the four distractor types, *F*(3,69) = 9.65, *p* < .001, $n_{p}^{2}$ = 0.3, justified a series of pairwise *t*-tests, corrected for multiple comparisons with the Bonferroni rule (α/6). These discrete tests indicated that participants were more often misidentifying direct gaze and upward gaze faces than other distractor types (for *p* values see Figure 2(c)).

**Table 1.** Average Error Rate in Main Experiment.

		Mean (%)	*SEM*
Target present trials	Errors: Misidentifications	33.2	3.38
	Errors: Incorrect rejections	24.09	2.75
Target absent trials	Errors: Misidentifications	44.01	3.81

Another one-way ANOVA (for repeated conditions) was used to analyze the target present trials and determine whether the average hit rate differed across the four target conditions (target direct gaze; target left gaze; target right gaze; target upward gaze; *F*(3,69) = 6.74, *p* < .001, $n_{p}^{2}$ = 0.23). The follow-up contrasts suggested that participants were, on average, more accurate (and a corresponding decrease in correct reaction time; *F*(3,69) = 15.51, *p* < .001, $n_{p}^{2}$ = 0.4) when the target was a direct gaze face rather than any other target type (Figure 2(d)).

## Experiment 2

**Figure 3.**
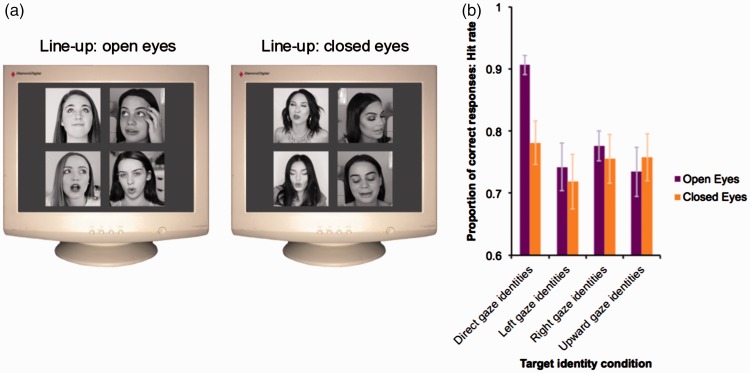
The procedure and results of Experiment 2. (a) Illustrative examples of the two lineup conditions in Experiment 2; The lineup arrays used in the open eyes condition were identical to those used in target present trials (Experiment 1). The lineup arrays in the closed eyes condition depicted the same target and distractor individuals as the lineups in the open eyes condition, different frames were sourced where all the tutors had their eyes closed. (b) Hit rate as a function of target condition (direct gaze face; left gaze face; right gaze face; upward gaze face) and lineup condition (open eyes; closed eyes). Error bars represent standard error of the mean.

The results of Experiment 1 suggest that participants recognizing faces in a lineup select more frequently faces making direct eye contact than faces looking elsewhere. One potential caveat in this design was viewpoint differences. Although we tried to capture stimuli that varied only in their gaze direction, it is possible that the earlier result reflects a bias toward front viewpoints, rather than direct gaze per se. One way to examine the contribution of gaze direction using the same stimulus set was to repeat Experiment 1 after removing gaze cues. Thus, in Experiment 2, we compared performance across two conditions; gaze cues present (*eyes open* condition) and gaze cues absent (*eyes closed* condition; see Figure 3(a)). If the results in Experiment 1 were driven by differences in gaze cues, one would expect no difference across the target conditions when the stimuli had their eyes closed.

### Method

#### Participants

In Experiment 2, we collected an independent sample of 16 participants (aged between 26 and 35; 11 participants were female). The sample size of 16 was determined by a power analysis of a previous study, thus, we recruited participants until we reached this number. All participants reported normal or corrected-to-normal vision. Although these participants were recruited from the same pool as described in Experiment 1, none of the participants were testing in Experiment 1.

#### Stimuli and procedure

We used the same equipment as in Experiment 1. The procedure for Experiment 2 was also identical to Experiment 1 except that there were no “target absent” trials and, thus, the participants were not given the option to reject the faces in the lineup. Instead, we forced them to select one of the four faces present (four-alternative forced choice task). The target-present trials were exactly the same; however, they were now referred to as the eyes open trials. Instead of having 96 target absent trials, as in Experiment 1, Experiment 2 had 96 eyes closed trials in Experiment 2 (see Figure 3(a)). The test stimuli from the eye closed trials were taken from the same videos that were used to collect the eyes open trials. We carefully selected the stimuli with closed eyes from blinks that occurred during those tutorials. Although there was no gaze information in these stimuli, we captured these blinks when the head was facing the front or turned to left, the right, or the ceiling (upwards) to mimic the corresponding “eyes open” stimuli. Due to the variability in the source material, the extent to which this was achieved differed greatly from trial to trial.

### Results

In the design, there were four levels of the variable “Target Type” (direct gaze vs. left gaze vs. right gaze vs. upward gaze) crossed with two levels of “Lineup” (eyes open vs. eyes closed) both were manipulated within participant. Therefore, we analyzed the average hit rate data using a 2 × 3 repeated measures ANOVA that yielded a main effect of Target Type, *F*(3,45) = 7.3, *p < *.001, $n_{p}^{2}$ = 0.33, but no evidence of a Lineup effect, *F*(1,15) = 2.03, *p = *.18, $n_{p}^{2}$ = 0.12. An interaction between these variables, *F*(3,45) = 3.4, *p* = .02, $n_{p}^{2}$ = 0.19; see Figure 3(b), motivated a set of four pairwise contrasts to compared performance between open eyes and closed eyes within each level of Target Type. These confirmed that closing the eyes only impacted the direct gaze target trials; participants were more accurate when the faces had their eyes open rather than closed (*p* = .001; α/4, cohen’s *d* = 1.06). This same difference was not significant for any other level of the target type (all *p* values > .0125; α/4).

The number of misidentification errors in the eight unique conditions was also investigated, as in Experiment 1. The same series of discrete paired *t*-tests (two-tailed) were used to determine whether closing the eyes changed the misidentification rate in any of the four gaze conditions (Bonferroni corrected for multiple comparisons; α/4). While this was true for the direct gaze condition, the misidentification rate was reduced when the stimuli had their eyes closed, compared with when they had their eyes open (*p* = .005, cohen’s *d* = 0.85), there was no evidence that this was true for the right (*p* = .07), left (*p* = .11), or upward gaze (*p* = .03) conditions.

## Experiment 3

One question that remained was whether recognition performance in Experiments 1 and 2 reflected an increase in identity sensitivity when faces were looking at the participants or a selection bias toward direct gaze faces. We addressed this question by running a third experiment to measure participant sensitivity to the identity signal without distractors.

### Method

#### Participants

An independent sample of 20 observers (12 female), all undergraduate psychology students at the University of Sydney, served as participants (age ranged between 22 and 31). A power analysis of a previous pilot investigating sensitivity across four visual conditions implied that a difference should emerge with a sample of size 9. All participants reported normal or correct-to-normal vision.

#### Stimuli and procedure

**Figure 4.**
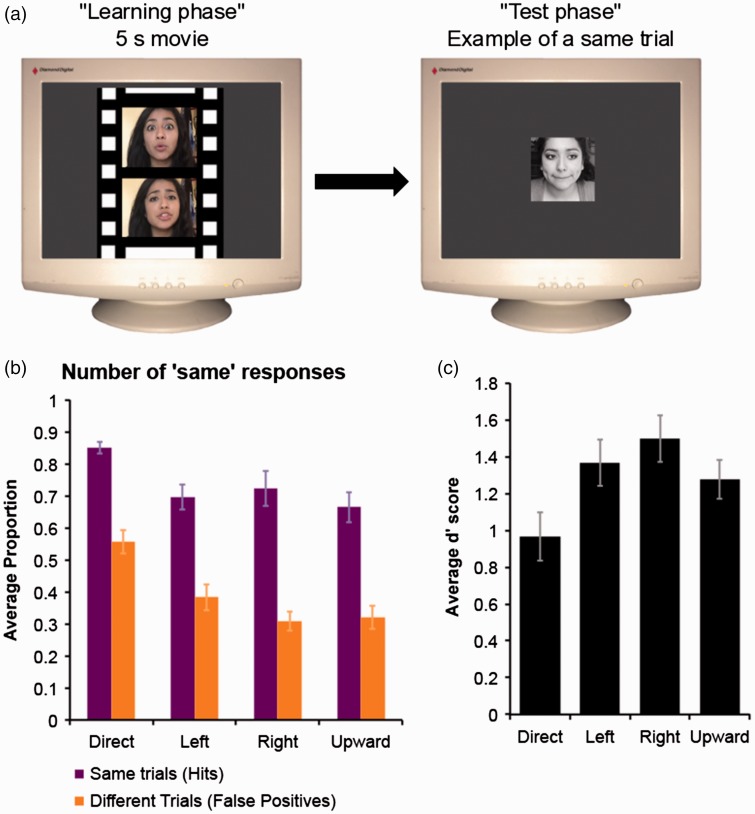
The sequential Same or Different Experiment. (a) Experimental procedure for Experiment 3. In this particular example, the correct answer would have been “same” because these faces all belong to the same person. (b) Average number of “same” responses in each trial type (same or different) as a function of face condition (direct gaze faces; left gaze faces; right gaze faces; upward gaze faces). Error bars = ± 1 *SEM*. (c) Average *d*′ scores (error bars = ± 1 *SEM*).

The procedure for the final experiment was the same as Experiment 1, except there was only one face presented in the test phase and participants simply indicated whether it was the same person they saw in the preceding movie or not (sequential same or different task). Target present trials were used as same trials (only the targets were visible in the test phase). Target absent trials were converted into different trials. We were careful to have 24 different trials with a direct gaze distractor, 24 with a left gaze distractor, 24 with a right gaze distractor, and 24 with a distractor looking up. Thus, in total, there were 96 same trials and 96 different trials. The timing parameters were the same as in Experiments 1 and 2 (see Figure 4(a)). The same equipment and testing room were used in all three experiments.

### Results

Overall accuracy was computed for each participant (% correct across all trials) and analyzed initially using a one-way ANOVA which yielded evidence of variation between the conditions, *F*(3,57) = 10.43, *p* < .001, $n_{p}^{2}$ = 0.35. To further investigate the a priori hypothesis regarding better performance in a specific condition (i.e., direct gaze), we ran a series of six discrete pairwise contrasts (two-tailed). There were three significant differences found in the accuracy data, after the correction necessary for multiple comparisons (α/6). The significant differences all indicated that participants were, on average, less accurate in the direct gaze condition (direct gaze vs. left gaze, *p* = .002, cohen’s *d* = 0.78; direct gaze vs. right gaze, *p* = .001, cohen’s *d* = 0.86; direct gaze vs. upward gaze, *p* = .001, cohen’s *d* = 0.85).

Inspection of Figure 4(b) suggested that participants were less accurate in the direct gaze condition because of an inflated false positive rate. The number of hits (correct responses in same trials) and false positives (incorrect responses in different trials) that each participant made across each of the four gaze conditions were transformed into a *d*′ score—these values were then averaged across participants. Sensitivity across gaze conditions was analyzed using a one-way ANOVA, *F*(3,57) = 5.71, *p* = .002, $n_{p}^{2}$ = 0.23. A set of six planned *t*-tests (two-tailed) yielded evidence that sensitivity differed between two discrete conditions only (direct gaze and right gaze, *p* = .008, α/6, cohen’s *d* = 0.66). None of the other pairwise comparisons were significant (all other *p* values > .09 after correction; see Figure 4(c)). A one-way analysis of variance of the corresponding response criterion values (c) indicated that there was a systematic change in the participant’s criteria across the four gaze conditions, *F*(3,57) = 32.61, *p* < .001, $n_{p}^{2}$ = 0.63. A set of planned *t*-tests (two-tailed) was used to find the source of the variance in the overall ANOVA. After a Bonferroni correction (α/6), only three discrete comparisons remained significant, the three that compared direct gaze to another gaze direction (direct gaze vs. left gaze, *p* < .001, cohen’s *d* = 1.35; direct gaze vs. right gaze, *p* < .001, cohen’s *d* = 1.51; direct gaze vs. upward gaze, *p* < .001, cohen’s *d* = 1.69). Together these results indicate that while sensitivity did not greatly differ across gaze conditions, the participants may have been using different criteria to make decisions in the direct gaze condition compared with the other gaze conditions. Certainly, we found no evidence to support the idea that performance with direct gaze targets is driven by increased sensitivity to the visual signal.

## General Discussion

The data across three experiments provide a clear indication that when the direction of gaze differs among the faces presented in a simultaneous lineup, faces looking directly at the participant are more likely to be selected. A large body of research has indicated that, for primates, detecting and understanding changes in the gaze direction of other people is a vital social skill (Baron-Cohen, 1995; Emery, 2000; Leonard et al., 2012; Tanaka & Sung, 2016). Until now, the widely held assumption was that gaze is important to read because it provides information about social intention. More simply put, the direction of a person’s gaze was thought to convey the direction of their current behavior or attention (Baron-Cohen, 1995; Calder et al., 2002; Tomasello & Carpenter, 2007). However, in studies of lie detection, it has been argued that gaze direction signals more about a person than the direction of their attention (Vrij et al., 2010; Vrij & Semin, 1996). Building on this, we hypothesized that gaze direction might interfere with a face recognition task and our results are largely consistent with this prediction. We argue that this might be because people find it difficult to recognize faces in a lineup (Bruce et al., 1999; Hancock et al., 2000; Megreya & Burton, 2006; Megreya et al., 2013) and they automatically process gaze direction information (Kuhn & Benson, 2007; Kuhn & Kingstone, 2009; Ricciardelli et al., 2002). Meaning that information about gaze direction is reliably available to participants, even when their instruction is to recognize a target face. Additionally, since gaze direction appears to be beneficial when participants are required to make old or new decisions (Adams et al., 2010; Farroni et al., 2007; Hood et al., 2003; Smith et al., 2006; Vuilleumier et al., 2005), it could well be the case they rely on this cue (automatically) in a lineup procedure as well.

The purpose of using a highly variable static stimulus set, together with the use of dynamic videos during the learning phase, was to increase the ecological validity of these findings, enabling us to draw rough inferences in a forensic context. However, a caveat we would like to consider is the nature of the static images we used in these experiments. They were highly variable because no attempt was made to standardize face size, face viewpoint, background information or principal visual characteristics, over and above luminance, and contrast (such as spatial frequency content; see Figure 1). Moreover, while we collected data regarding the perceived gaze direction of the faces comprising the “direct gaze” condition, the stimuli in other gaze direction conditions remain subject to a potential researcher bias. For instance, we have no evidence that the faces in the “left gaze” condition are perceived as looking left to anyone other than ourselves. For this reason, perhaps it would be better to think of them as gaze deviants (i.e., not making direct eye contact) rather than three discrete categories (left gaze; right gaze; upward gaze). Certainly, this phenomenon could be confirmed with more a standardized stimulus set, where head position (i.e., viewpoint) and gaze direction could be manipulated independently. Also, we acknowledge that the experience of attending a real life lineup is different in nature (one viewing vs. multiple ones) and outcome (legal repercussions) to our experimental set up. Therefore, after having established the occurrence of the effect in a laboratory setting, it is essential to establish if the reliance of eye gaze as a heuristic in the face of uncertainty is still present in the same magnitude under a more ecologically valid conditions.

## Conclusion

Based on the present literature, it seems that gaze direction has been overlooked as a potential problem when asking witnesses to identify a person in a lineup (either live viewing or in a photo array). Although the current procedure may encourage people to look forward, the brain is incredibly sensitive to direct eye contact (Baron-Cohen, 1995; Calder et al., 2002; Pelphrey et al., 2005; Pelphrey et al., 2004) and, thus, if no effort is made to hold gaze direction, constant small variations can be expected among people in a photo lineup. Importantly, these results suggest that people are biased toward selecting direct gaze faces when gaze direction differs among faces, inflating the misidentification rate. It is a simple physical attribute that, with very little effort, can be standardized among a group of people and, thus, neutralized as a contributing factor to misidentification error.
